# Supplementary material for: Room temperature nondestructive encapsulation via self-crosslinked fluorosilicone polymer enables damp heat-stable sustainable perovskite solar cells
Source: Nat Commun. 2023 Mar 11;14:1342. doi: 10.1038/s41467-023-36918-x (PMC10008636; doi:10.1038/s41467-023-36918-x)
Supplement: Supplementary file 1 — Supplementary Information [file 41467_2023_36918_MOESM1_ESM.pdf]

SUPPLEMENTARY INFORMATION

Room temperature nondestructive encapsulation via self-crosslinked fluorosilicone polymer enables damp heat-stable sustainable perovskite solar cells

Tong Wang[1], Jiabao Yang[1], Qi Cao[1], Xingyu Pu[1], Yuke Li[2], Hui Chen[1], Junsong Zhao[1], Yixin Zhang[1], Xingyuan Chen[1], Xuanhua Li[1]*

[1] State Key Laboratory of Solidification Processing, Center for Nano Energy Materials, School of Materials Science and Engineering, Northwestern Polytechnical University, Xi´an 710072, China

[2] Department of Chemistry and Centre for Scientific Modeling and Computation, Chinese University of Hong Kong, Shatin, Hong Kong, China

* Mail address: lixh32@nwpu.edu.cn

This PDF file includes:

Supplementary Methods
Supplementary Figures 1 to 36
Supplementary Tables 1 to 8
Supplementary Note 1
Supplementary References 1 to 5

Supplementary Methods

Synthesis of NiO_x

A green solution was produced by dissolving Ni $(NO_3)_2 \cdot 6H_2O$ (0.5 mol) in deionized water (100 mL) under magnetic stirring for 5 min. After that, add NaOH (10 mol L^{-1}) solution gradually until the pH of the Ni $(NO_3)_2$ solution reaches 10. After 10 min, the reaction solution was centrifuged, followed by numerous washes with deionized water. The prepared product was dried in a vacuum at 80 °C for 12 h and calcined at 270 °C for 2 h to obtain NiO_x nanoparticles.

Synthesis of TiO_2

To prevent local ethanol warming, 2 mL $TiCl_4$ was slowly injected into 10 mL of cold anhydrous ethanol while being vigorously stirred. The mixture was added to 70 mL of anhydrous benzyl alcohol in a 250 mL 3-neck round bottom flask after the solution had cooled to room temperature. Then, the flask was capped and stirred in an oil bath at 90 °C for 6 h until the mixture became ivory-white. The TiO_2 nanocrystals were centrifuged after being precipitated from the original solution by the addition of ethanol. The solid was washed by adding ethanol and repeated twice. Then the produced white precipitate was dispersed in chloroform with a concentration of 40 mg mL^{-1}.

Fabrication of $CsPbI_2Br$-based PSCs

The FTO glasses (1.5 cm×1.5 cm) were cleaned with deionized water, acetone, and ethyl alcohol. The substrates were spin-coated with NiO_x (20 mg mL^{-1}) for 30 s at 3000 rpm after being treated with UV ozone plasma for 15 min. The substrates were then heated at 100 °C for 10 min. The $CsPbI_2Br$ perovskite precursor solution by mixing CsI, $PbBr_2$, and PbI_2 (molar ratio = 2:1:1) in DMSO/DMF (1/4 V/V). Spin-coating of the precursor solution onto NiO_x film was performed at 1000 rpm for 15 s and 4000 rpm for 45 s. 150 μL CB was added during the last 20 s. The films were annealed for 2 min at 50 °C and for 10 min at 160 °C. In the following step, the electron transport layer TiO_2 (45 mg in 1 mL chloroform) was spin-coated on the perovskite film at 3000 rpm for 50 s and heated at 160 °C for 10 min. Finally, vacuum evaporation equipment was used to evaporate the Al (100 nm) at 2×10^{-6} mbar. The effective area of the device was 0.1 cm^2.

Fabrication of $MAPbI_3$-based PSCs

The FTO glasses (1.5 cm×1.5 cm) were cleaned with deionized water, acetone, and ethyl alcohol. In the following step, the electron transport layer TiO_2 (45 mg in 1 mL chloroform) was spin-coated on the perovskite film at 3000 rpm for 50 s and heated at 160 °C for 10 min. The $MAPbI_3$ precursor solution was obtained by mixing MAI and PbI_2 (molar ratio = 1:1) in γ-butyrolactone and DMSO (7/3

V/V). The perovskite solution was spin-coated onto TiO_2 layer at 1000 rpm for 10 s and 3000 rpm for 60 s. 180 μL CB was added after the last 35 s. Then the perovskite films were annealed at 100 °C for 10 min. The PTAA solution containing PTAA (30 mg in 1 mL chlorobenzene) and TPFB (3 mg) was used as the hole transport layer. The solution deposited the perovskite film at 2000 rpm for 30 s. Finally, vacuum evaporation equipment was used to evaporate the Au (100 nm) at 2×10^{-6} mbar. The effective area of the device was 0.1 cm^2.

Fabrication of CsMAFA-based perovskite solar modules

The FTO glasses (5 cm × 5 cm) were pre-patterned with the laser and cleaned with deionized water, acetone, and ethyl alcohol. The as-patterned FTO glasses were followed by depositing the 40-50 nm TiO_2 layer using the spin-coating method. Then, blade coating (the speed of 3 mm s^{-1}) was used to coat the perovskite precursor solution onto the TiO_2 layer and the N_2 knife was operated at a pressure of 20 psi. A distance of 150 μm (z =0.15 mm) separated the blade-coater and substrates. A final annealing step at 100 °C for 20 min was performed on the perovskite films. PTAA solution was used as the hole transport layer for the thermal stability test. We used the vacuum evaporation equipment to evaporate the Au (100 nm) at 2×10^{-6} mbar. Two laser scribing procedures were used to complete module construction: before and after electrode deposition. The effective area of the device was 15.8 cm^2.

Quantum Chemical Calculations

Density functional theory (DFT) calculations were performed via Gauss 09 program using the B3LYP functional and the all-electron double-ξ valence basis sets with polarization functions 6-31G* (B3LYP/6-31G*). Geometry optimizations of functional groups were carried out under the condition that all atoms were completely relaxed, without the influence of solvent.

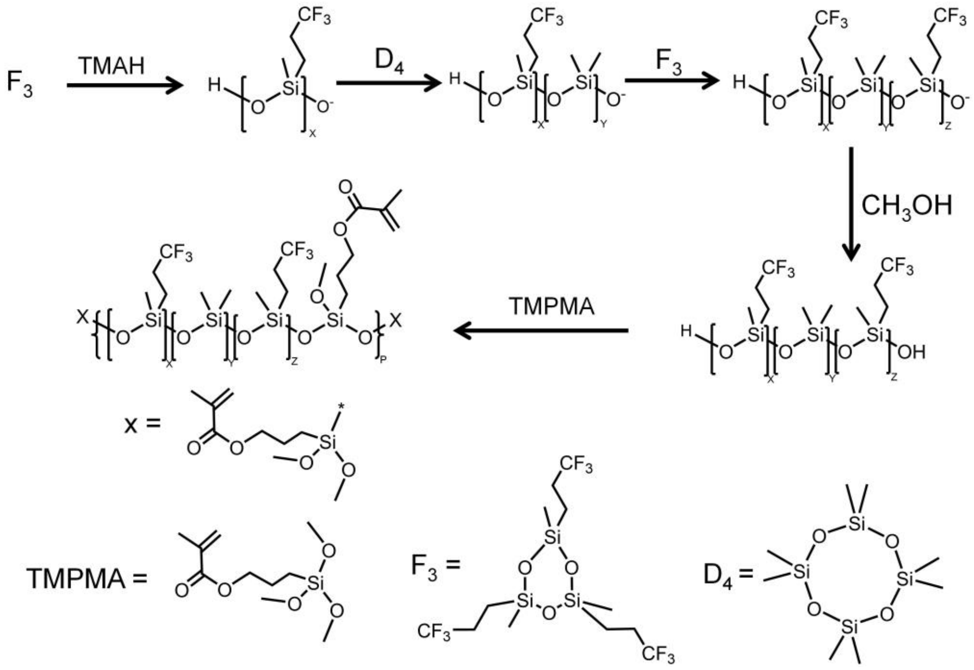

Supplementary Figure 1. The synthetic route of the fluorosilicone polymer FDP. In the scheme, we show the synthesis process of the FDP. First, fluorosilicone block copolymers are synthesized by ring-opening polymerization reaction of monomers F_3 and D_4. Then, the block copolymers and small molecular monomers TMPMA are generated the reaction product FDP via hydrolysis and condensation reactions.

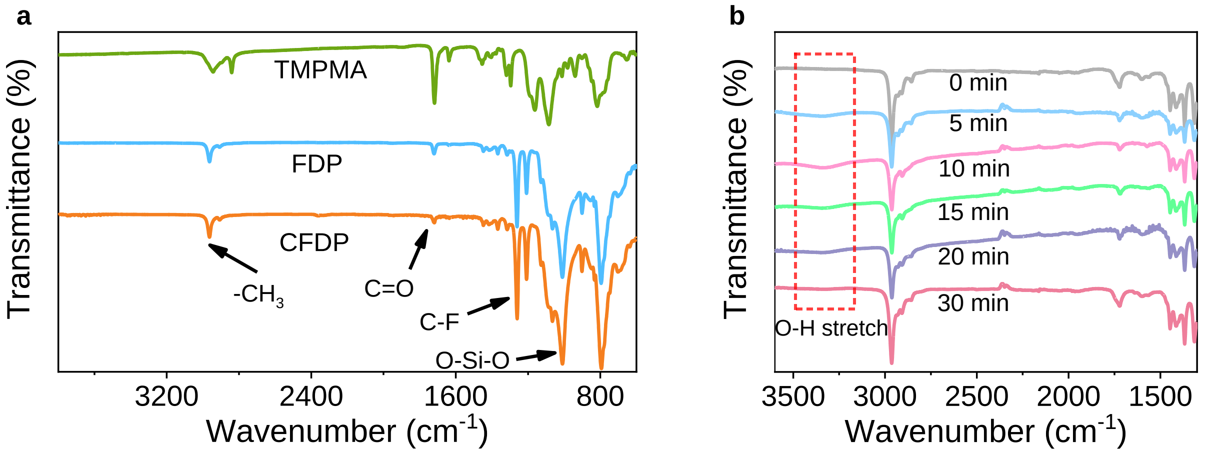

Supplementary Figure 2. (a) FTIR characterization of TMPMA, FDP, and CFDP. (b) FTIR characterization of the FDP at various times during the condensation reaction. We employed the FTIR spectra to make a judgment on the structure of FDP. The stretching vibration peak of $-CH_3$ is observed at 2962 cm^{-1}. The peak at 1720 cm^{-1} is the stretching vibrations of C=O, while the stretching vibration peaks of O-Si-O are observed at 1011 cm^{-1}. The peak at 793 cm^{-1} is the stretching vibrations of $Si-(CH_3)_3$. There is a peak that appeared at 1261 cm^{-1}, which corresponds to the C-F groups.[1] We observed the presence of alcoholic hydroxyl group during the condensation reaction (**Supplementary Figure 2b**), indicating that the production of side product (alcohol) and outgassing during the reaction.

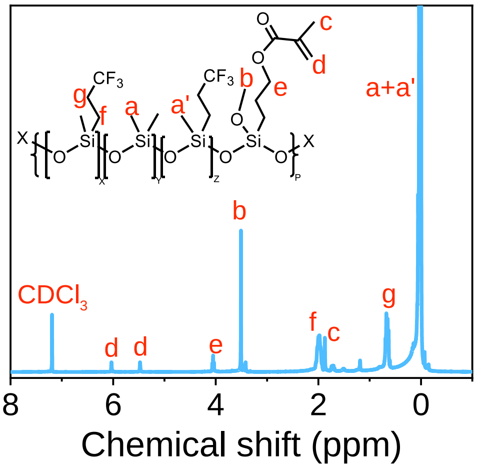

Supplementary Figure 3. ^{1}H NMR spectra of the FDP polymer. We performed ^{1}H-NMR to confirm our proposed reaction products for FDP. The chemical shift at 0.02 ppm corresponds to Si-CH_3, the chemical shift at 3.51 ppm is -$SiOCH_3$, the chemical shift at 1.87 ppm is CH_3- bonded to C=C, the chemical shift at 6.03 ppm and 5.47 ppm is CH_2=C-, and the chemical shift at 4.04 ppm is -CH_2- group bonded with oxygen. The chemical shift at 2.00 ppm and 0.67 ppm belongs to the -CH_2- groups and -CH_2CF_3. These characteristic chemical shifts suggest that the functional groups -$SiOCH_3$ and -CH_2CF_3 are successfully introduced into the polymer structure.

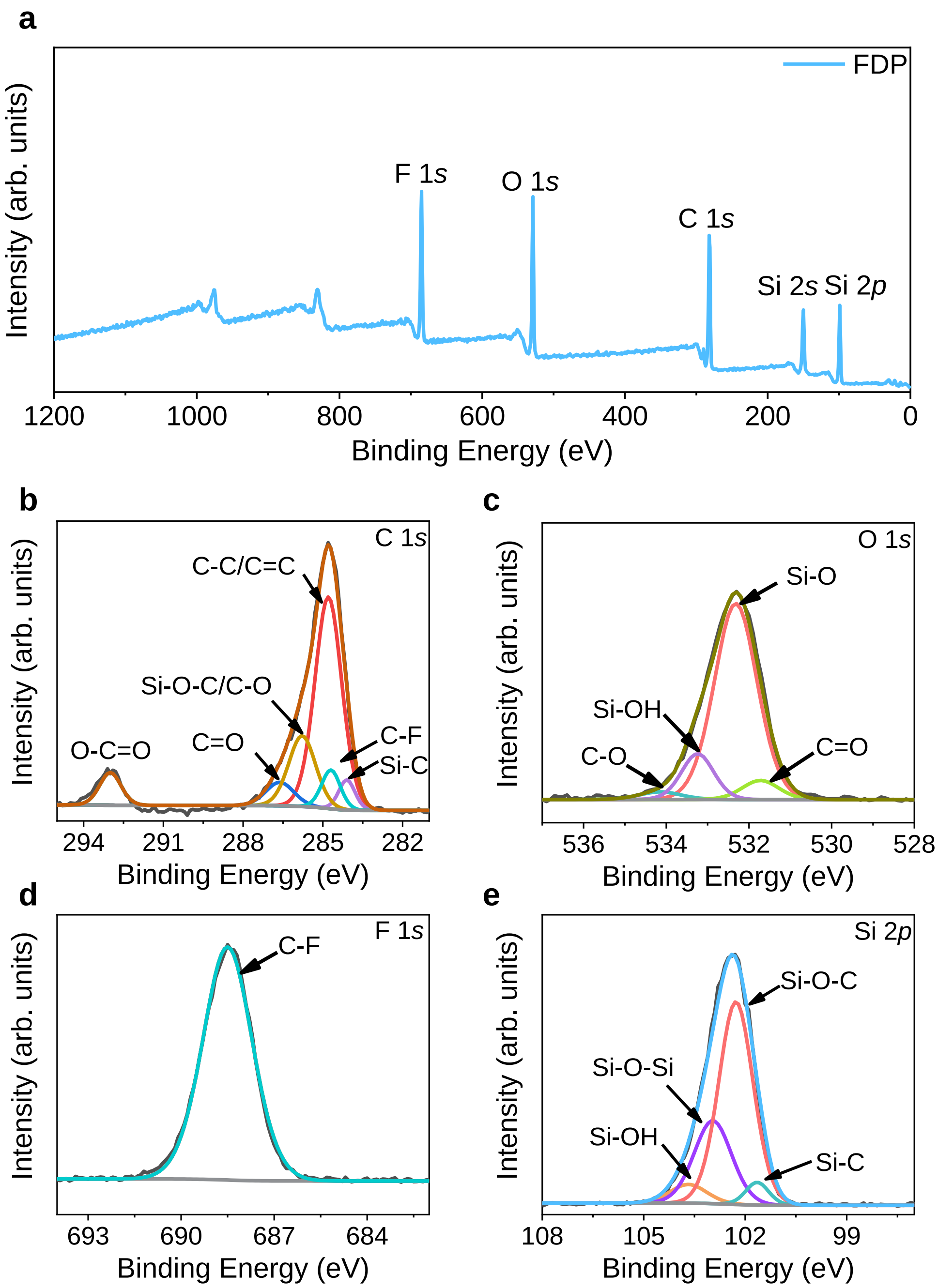

Supplementary Figure 4. XPS spectra of (a) full spectra of FDP, (b) the C 1*s* spectra of FDP, (c) the O 1*s* spectra of FDP, (d) the F 1*s* spectra of FDP, and (e) the Si 2*p* spectra of FDP. We characterized the XPS spectra of FDP. The full spectra of the FDP show the peaks of C, O, F, and Si element, revealing the successful synthesis of FDP. We fitted the C 1*s*, O 1*s*, F 1*s*, and Si 2*p* spectra and analyzed the functional groups in the FDP. The results are shown in **Supplementary Figure 4**.

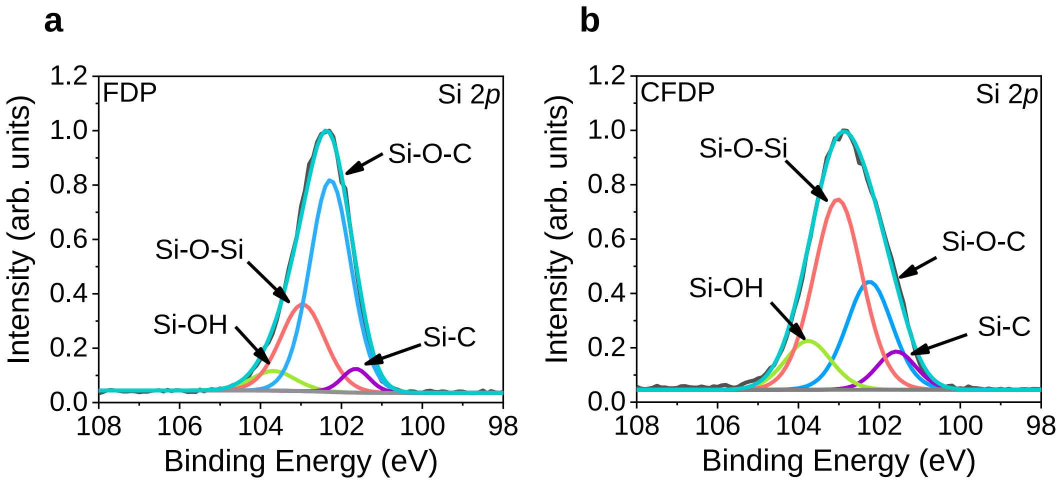

Supplementary Figure 5. The normalized XPS Si 2*p* spectra of (a) FDP and (b) CFDP. There are four main peaks in the spectra of Si 2*p* in the FDP and CFDP films. The peak at 102.9 eV is assigned to Si-O-Si, while the peak at 102.3 eV refers to Si-O-C, and the other peaks at 101.7 eV and 103.7 eV are ascribed to Si-C and Si-OH.[2]

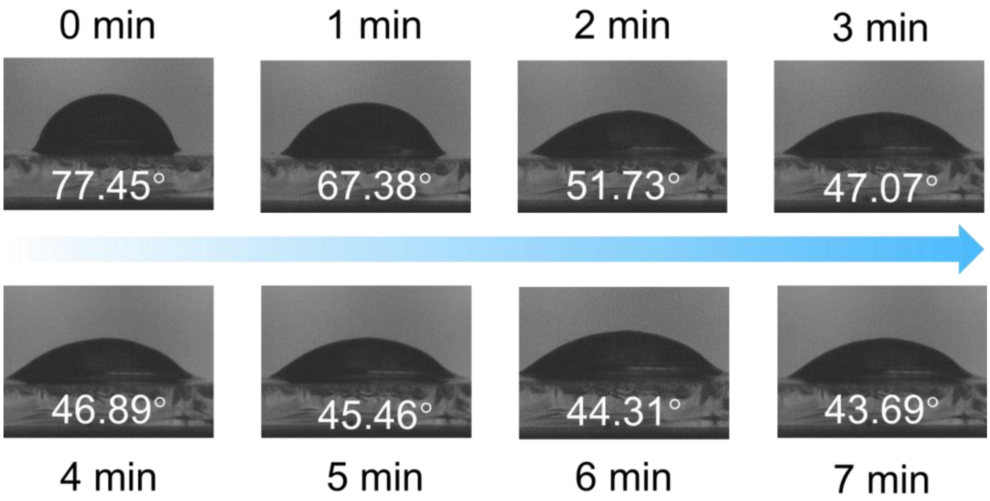

Supplementary Figure 6. The water contact angles of FDP film as a function of time.

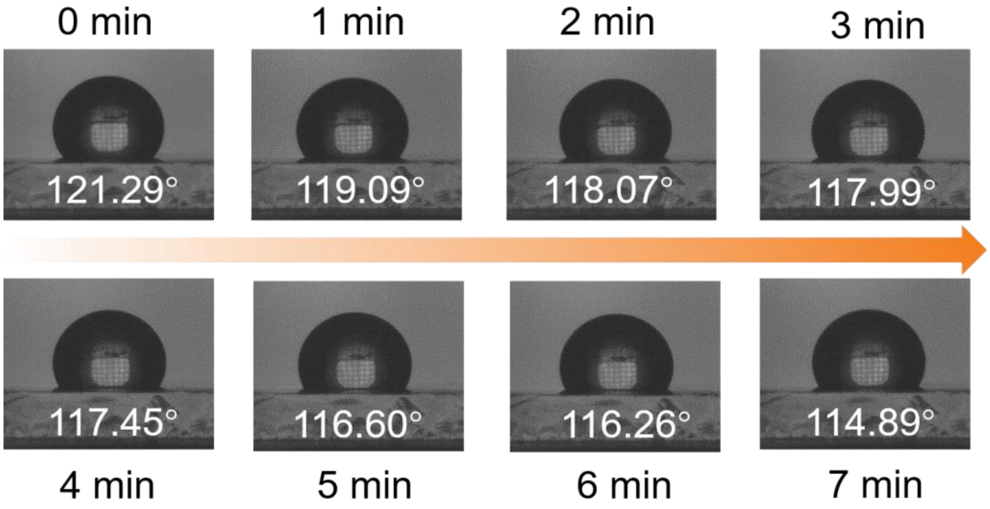

Supplementary Figure 7. The water contact angles of CFDP film as a function of time.

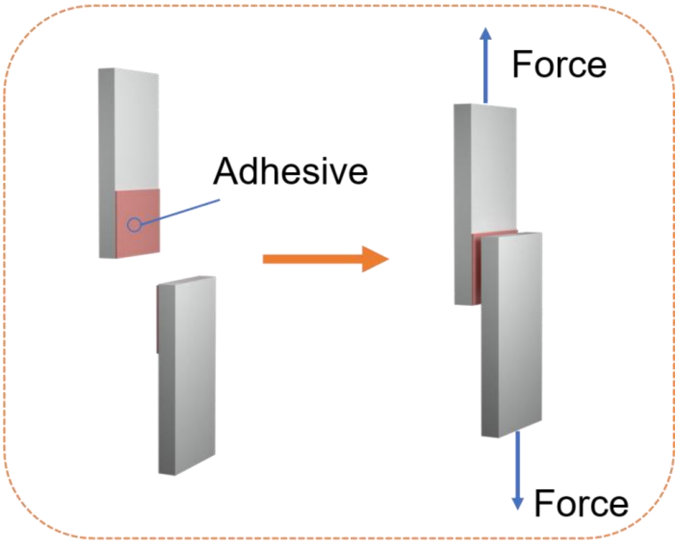

Supplementary Figure 8. Scheme of the single-lap tensile shear strength test. The size of aluminum sheet sample is 50 × 12 mm^2 and the size of bonded area size is 12 × 6 mm^2. The thickness of the adhesive layer is about 200 μm. The test speed is 5 mm min^{-1}. Two aluminum sheets are bonded together with the FDP and CFDP. Then, the aluminum sheets are stretched in opposite directions via the stretching equipment until the two specimens are pulled to failure.

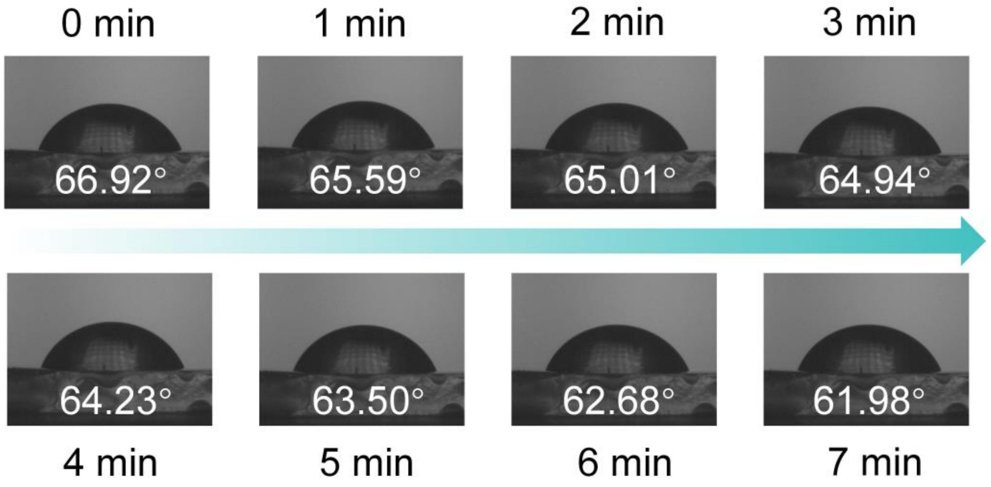

Supplementary Figure 9. The water contact angles of UV resin film as a function of time.

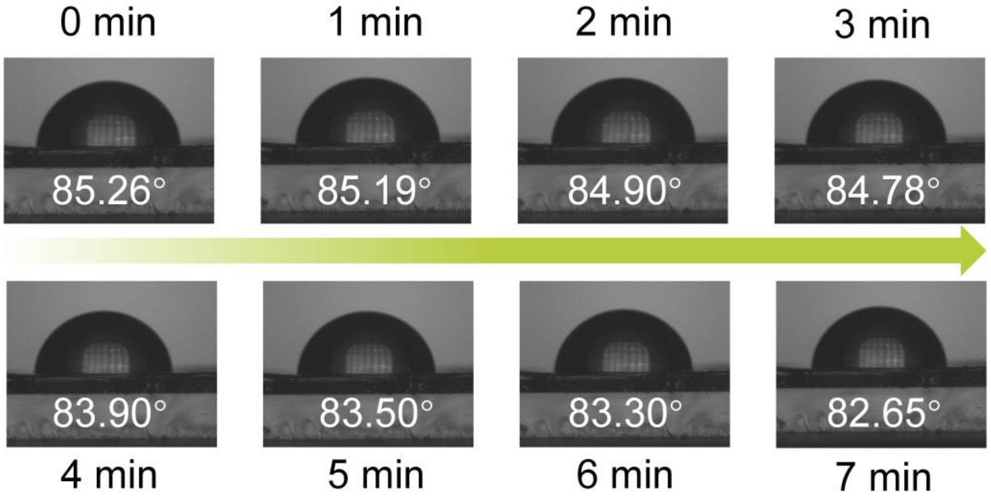

Supplementary Figure 10. The water contact angles of POE film as a function of time.

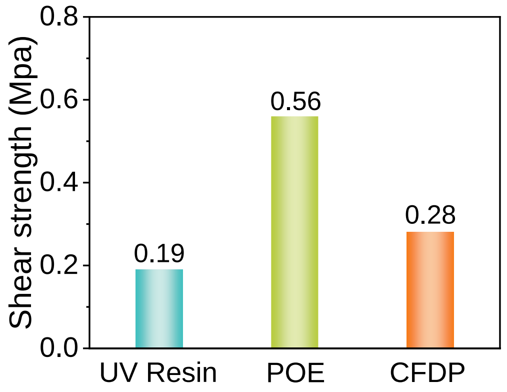

Supplementary Figure 11. The adhesion strength of UV resin, POE, and CFDP. The adhesion strength is calculated via the following formula: $\tau = {}^{F}\!/_{A}$, τ is the adhesion strength, F is the maximum load, and A is the bonding area. The adhesion strength of UV resin and POE is 0.19 Mpa and 0.56 Mpa, while the adhesion strength of CFDP is 0.28 Mpa.

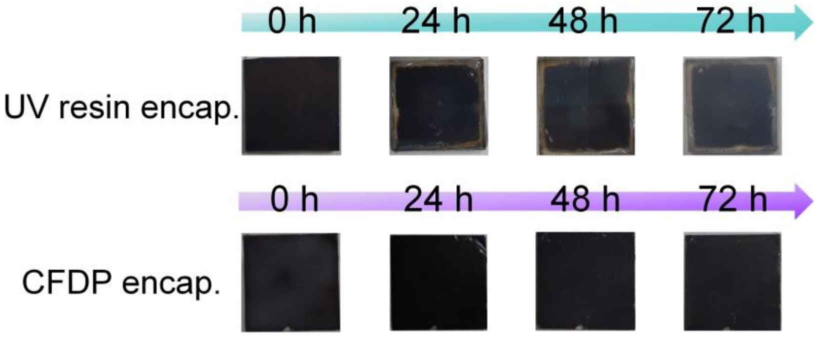

Supplementary Figure 12. The images of perovskite films with UV resin encapsulation and CFDP encapsulation under different aging times. The edges of the UV resin encapsulated perovskite film has already started to decompose right after 48 h in **Supplementary Figure 12**. This might be because the outgassed vapor of UV resin damages the perovskite film during the UV-curing process.[3] In contrast, the perovskite film with CFDP encapsulation shows excellent compatibility with the polymer gel during the curing process.

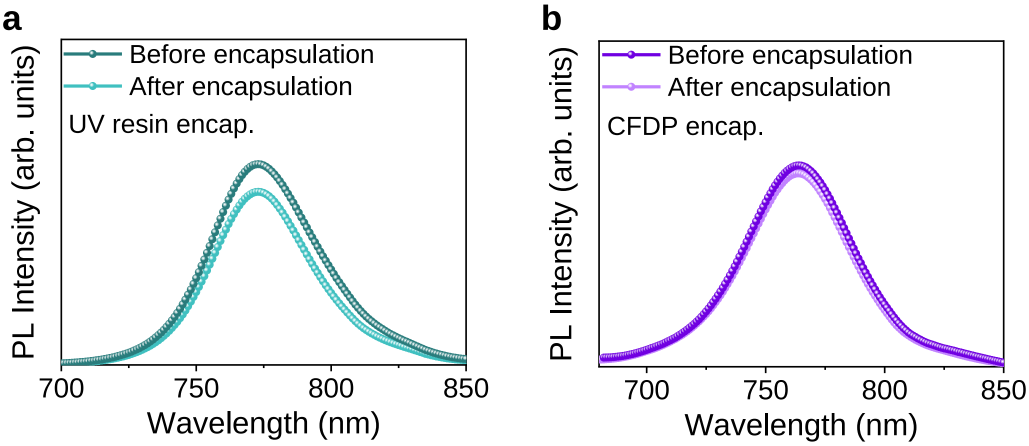

Supplementary Figure 13. Steady-state PL spectra of perovskite films before and after (a) UV resin encapsulation and (b) CFDP encapsulation. We conducted steady-state PL spectra of the perovskite films before and after encapsulation. The perovskite films with UV resin encapsulation show a reduced emission intensity. Contrastingly, perovskite films with CFDP encapsulation demonstrate more robust emission peak intensity with the same level of intensities at 764 nm.

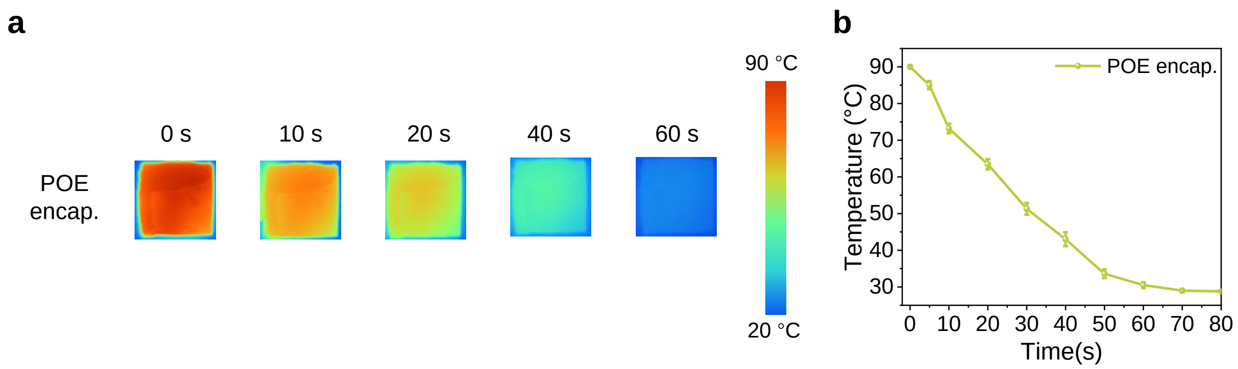

Supplementary Figure 14. (a) IR thermal images of POE encapsulated perovskite films during a cooling test. (b) The surface temperature change of perovskite films based on POE encapsulation as a function of time. The error bars represent the standard deviation for three samples.

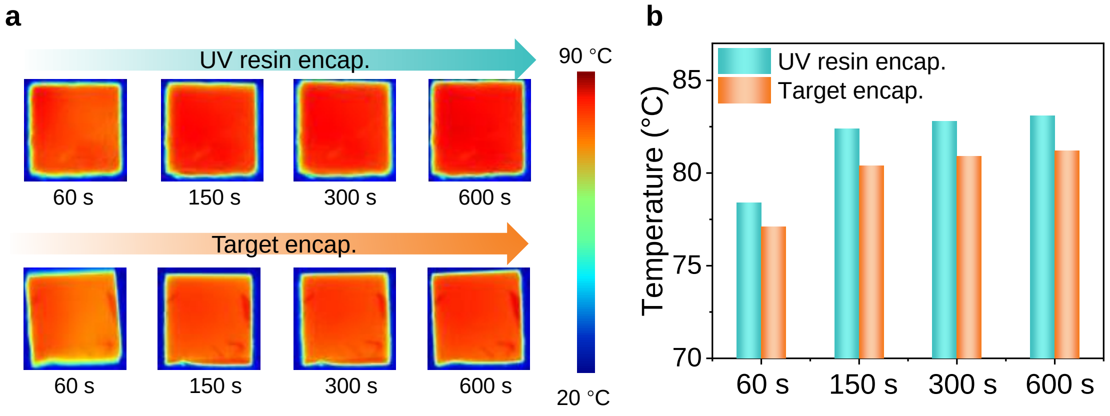

Supplementary Figure 15. (a) IR thermal images of UV resin and target encapsulated perovskite films under thermal stress. (b) Statistical histogram of the average temperature. The encapsulated sample reaches the thermal equilibrium state when the average temperature reaches the stable value.

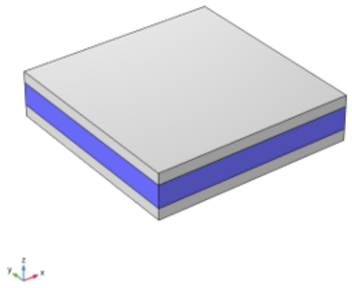

Supplementary Figure 16. The model of encapsulated perovskite film in finite element simulation. To simplify the calculation, we constructed the simple model of the encapsulated perovskite film with three floors. The grey layers represent the polymer gel and the blue layer represents the perovskite film.

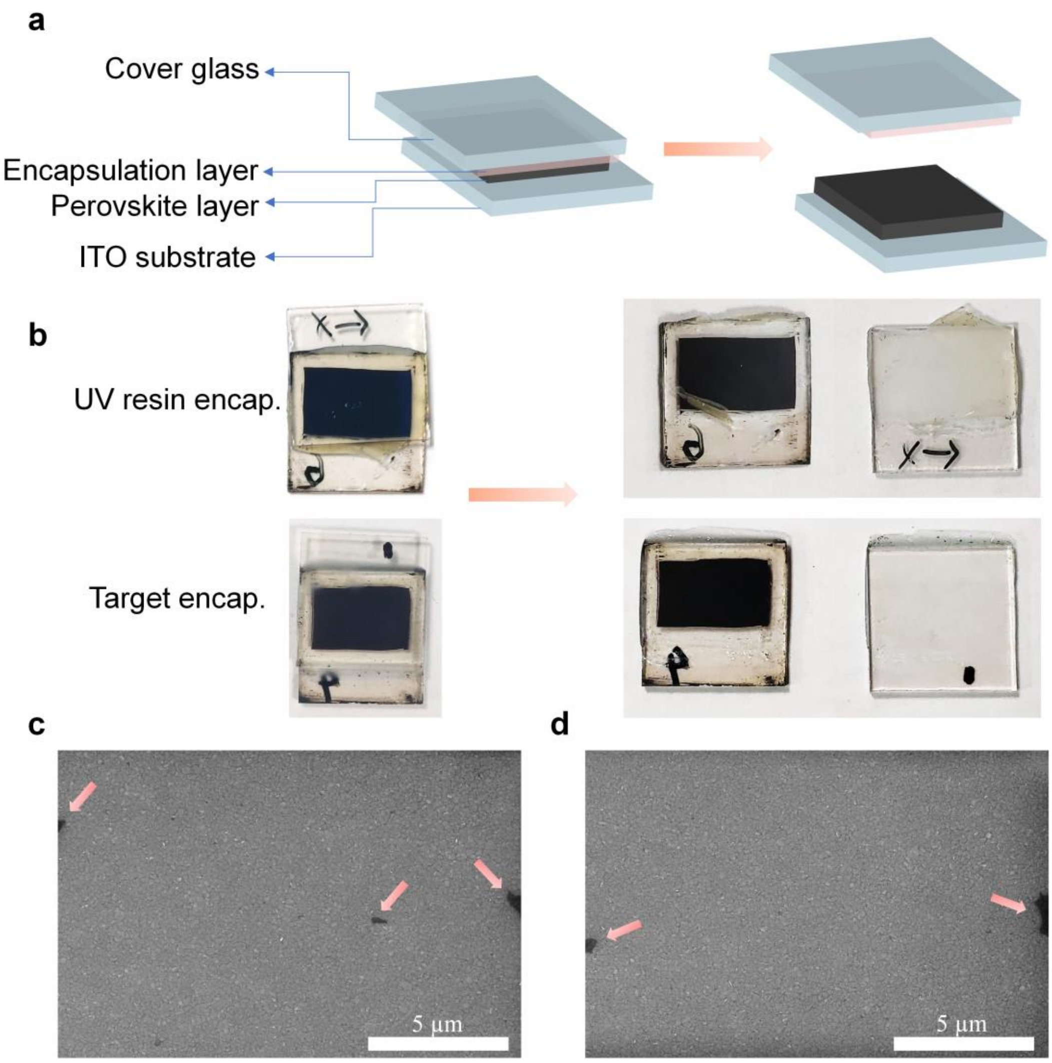

Supplementary Figure 17. (a) Schematic diagram of removing the encapsulation for preparing the SEM samples. (b) Photographs of the encapsulated perovskite films before and after the encapsulation removal. Top-view SEM images of perovskite films after (c) UV resin encapsulation and (d) target encapsulation removal. The pink arrow points to the holes. To better remove the cover glass and encapsulation layer, we used half of the glass to cover the perovskite films instead of the previous full coverage. We chiseled encapsulated glass and encapsulant with a scalpel carefully in **Supplementary Figure 17a-b**. We observed that there were a small number of holes on the surface of UV resin and target encapsulated perovskite films in **Supplementary Figure 17c-d**. To ensure the accuracy of the experiment, we selected the part of the film without obvious damage for comparison to better reveal the effect of heat dissipation capability on the encapsulated perovskite films, while excluding the influence of removing the encapsulation process on the perovskite films.

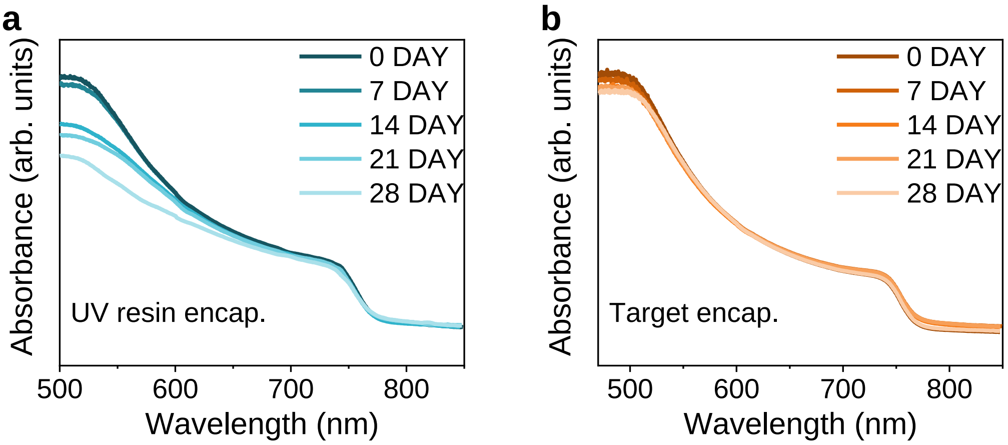

Supplementary Figure 18. UV-vis absorption spectra of perovskite films with (a) UV resin encapsulation and (b) target encapsulation after age in the air at 85 ºC at different times.

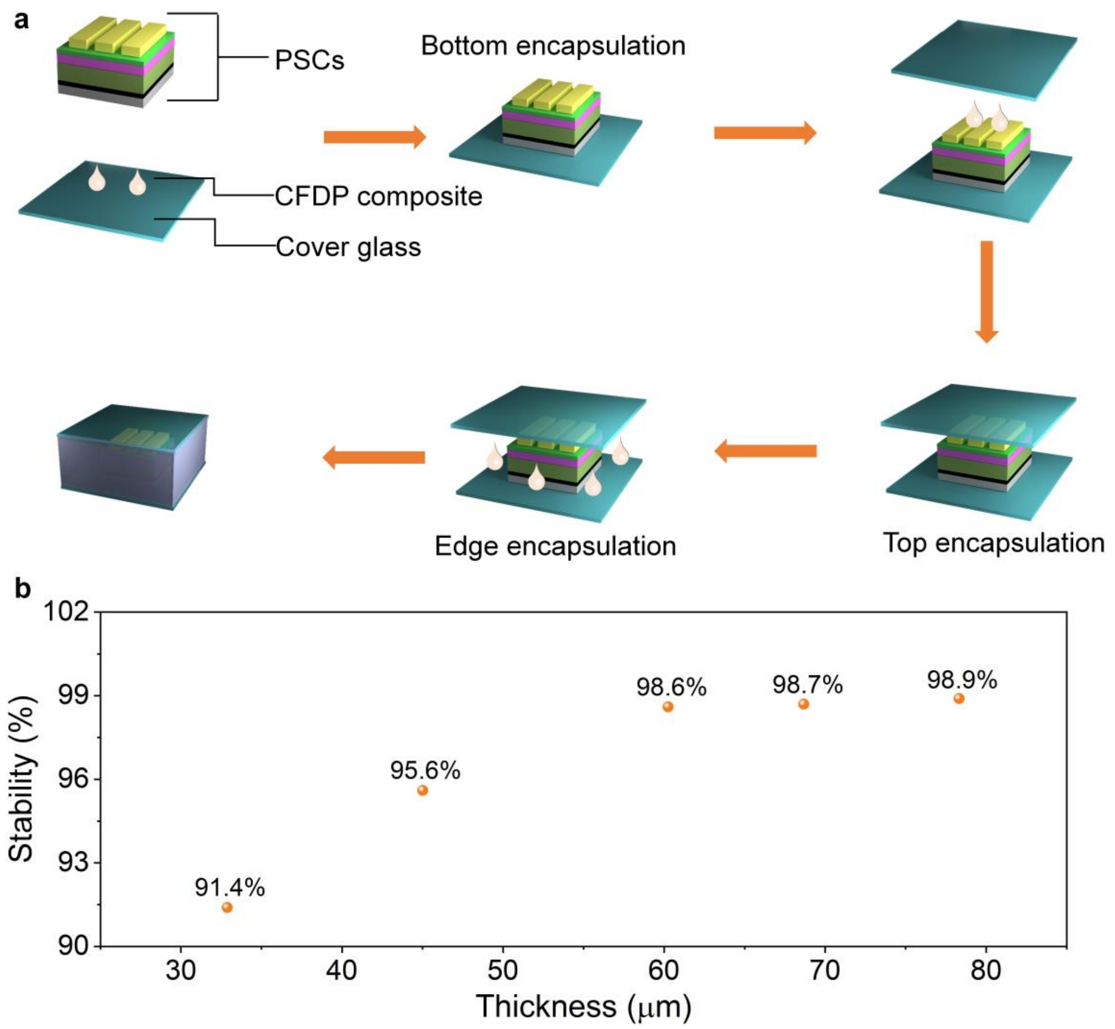

Supplementary Figure 19. (a) Scheme of the target encapsulation process. (b) Relationship between the thickness of encapsulant and the stability of encapsulated devices. The encapsulated devices are aged at maximum power point under AM 1.5 illumination at 55±5 ºC in the air for 100 h. The ordinate represents the normalized efficiency of the encapsulated devices after aging compared to the initial efficiency.

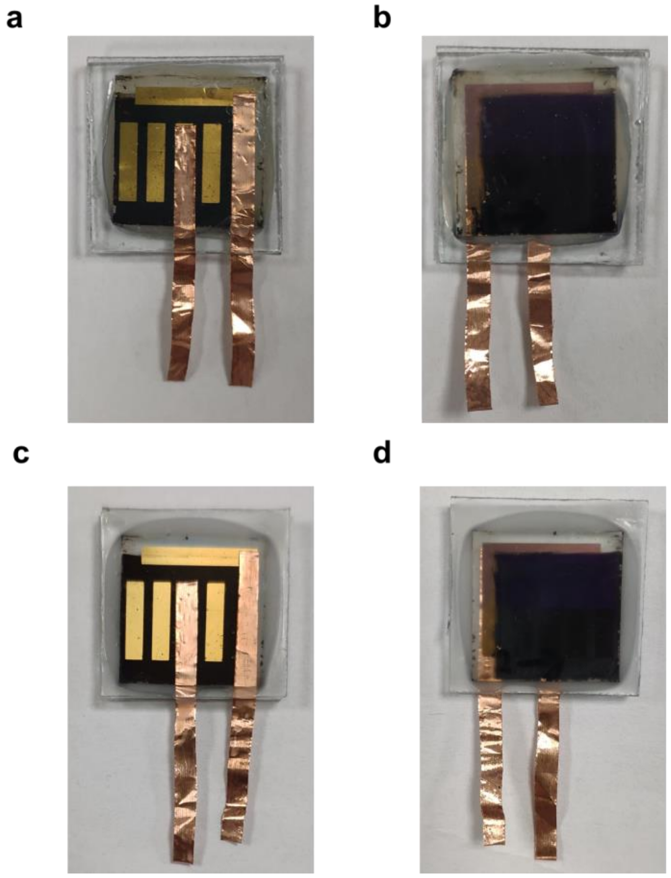

Supplementary Figure 20. The two-sided photo-image of the device is encapsulated by UV resin of (a) and (b). The two-sided photo-image of the device is encapsulated by CFDP composite of (c) and (d).

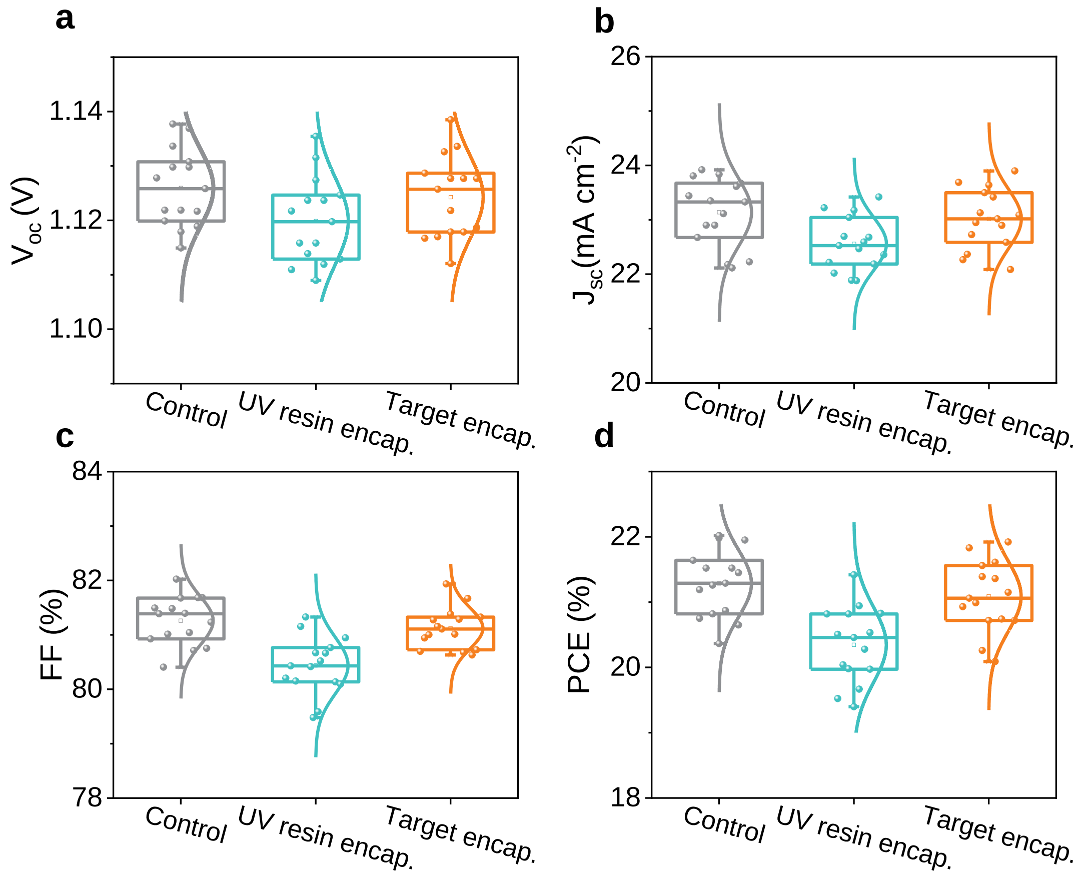

Supplementary Figure 21. The statistical distribution of (a) V_{OC}, (b) J_{SC}, (c) FF, and (d) PCE for control, UV resin encapsulation, and target encapsulation (15 devices were collected from the different batches). The center line represented median, and the top and bottom box limits represented the upper and lower quartiles, respectively. The small rectangle represented the mean value. The maximum/minimum values are represented by the top/bottom bars.

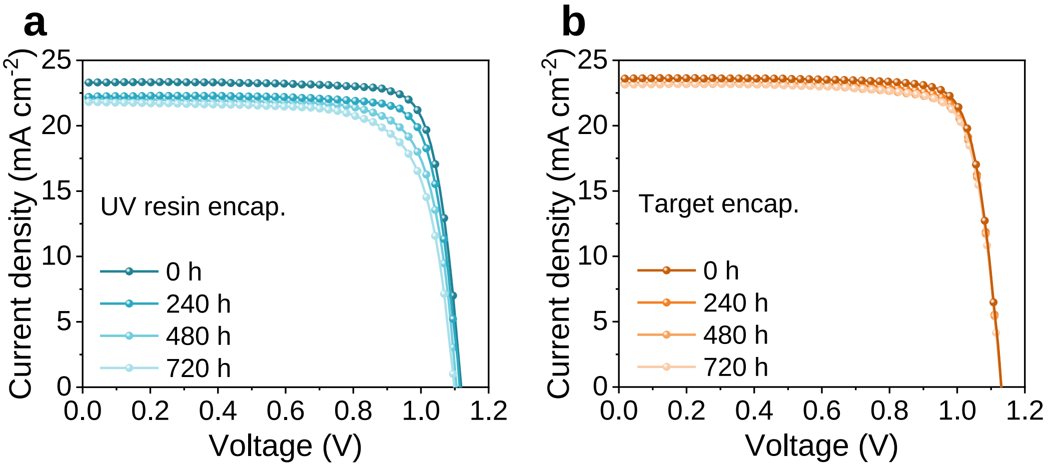

Supplementary Figure 22. The attenuation curve of PCE of encapsulated devices with (a) UV resin encapsulation and (b) target encapsulation in a nitrogen glove box at 85 ºC.

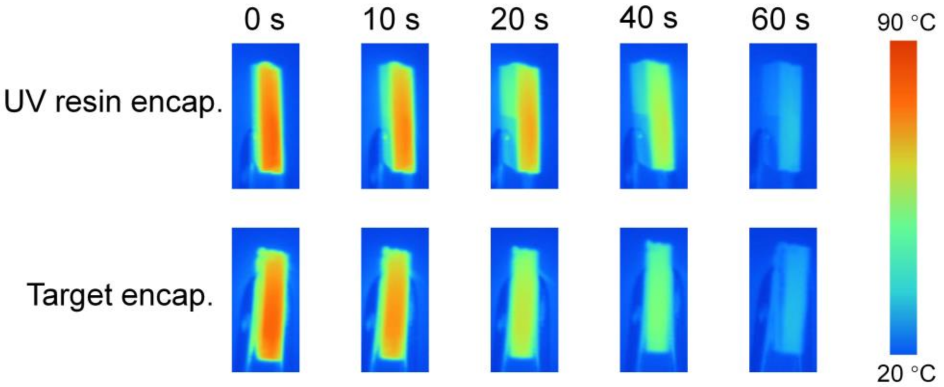

Supplementary Figure 23. IR thermal images of encapsulated PSCs under a cooling test.

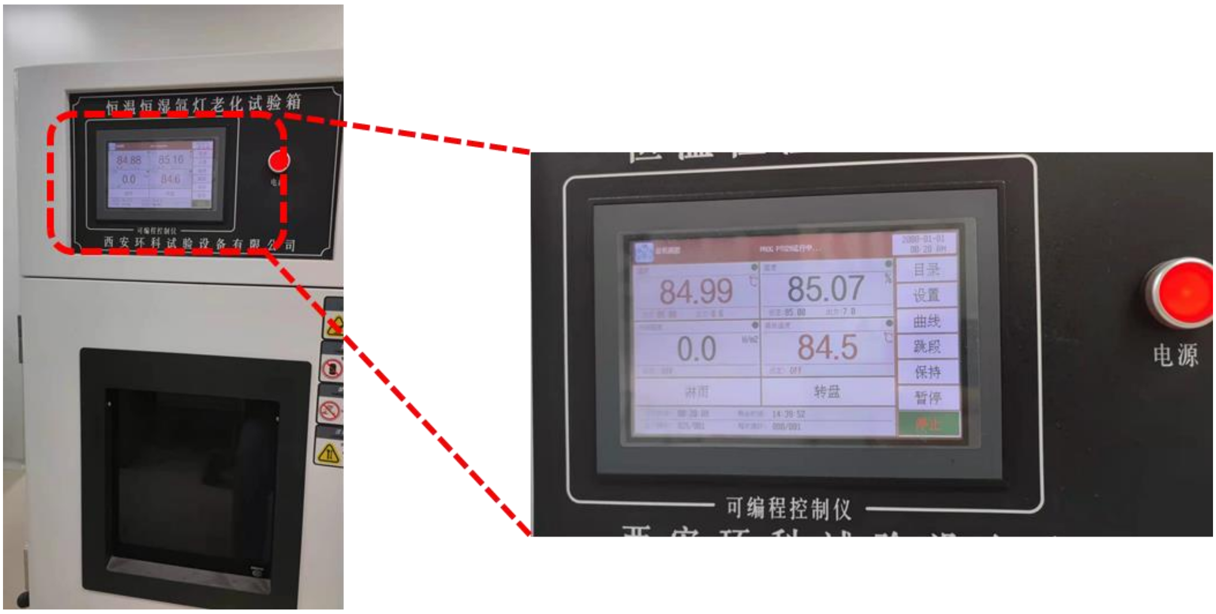

Supplementary Figure 24. The photographs of the damp-heat chamber. The relative humidity and temperature were maintained at 85±0.5% and 85±0.5 °C, respectively, following International electrotechnical commission (IEC) 61215:2016 standards.[4]

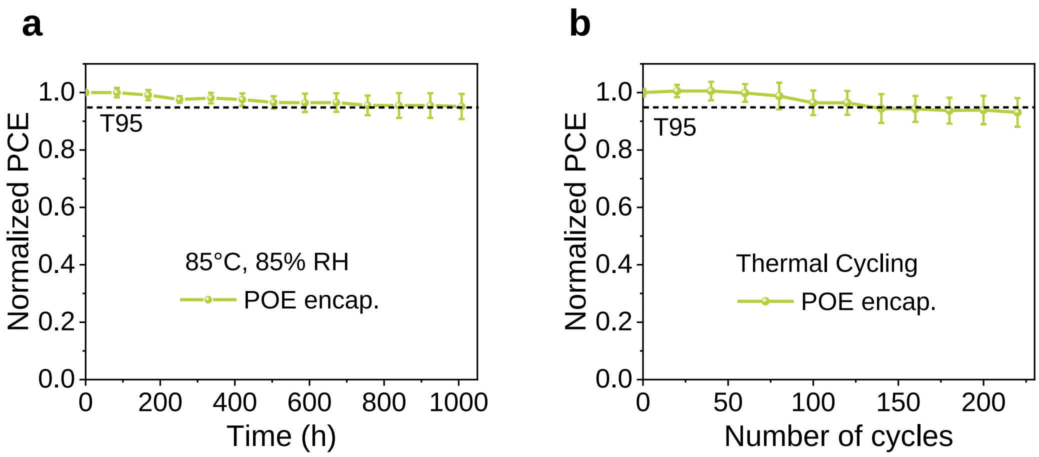

Supplementary Figure 25. Efficiency evolution of PSCs under (a) damp heat test at 85 ºC and 85% relative humidity and (b) thermal cycling test from −40 ºC and 85 ºC. Error bars represent the standard deviations from the statistical results of the three devices. The epoxy resin is used for edge sealing for better stability. The devices with POE encapsulation retained 95% of their initial PCE values after 1000 h in the damp heat test and retained 93% of their initial efficiency after 220 cycles in the thermal cycling test.

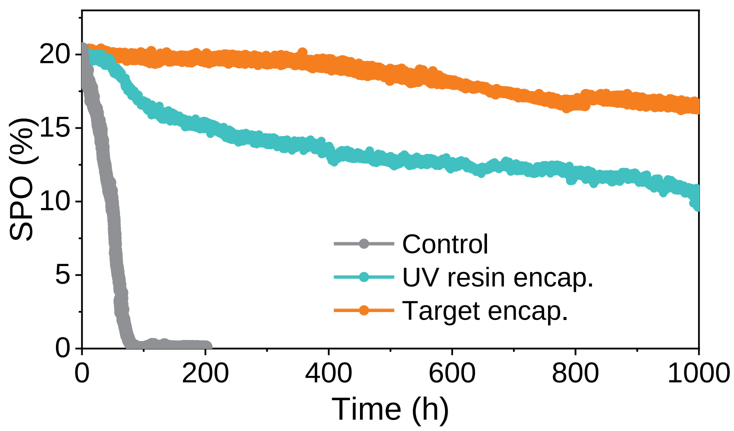

Supplementary Figure 26. The SPO attenuation curve of control, the device with UV resin encapsulation, and the device with target encapsulation at maximum power point under AM 1.5 illumination at 55±5 ºC in the air. The epoxy resin is used for edge sealing for better stability.

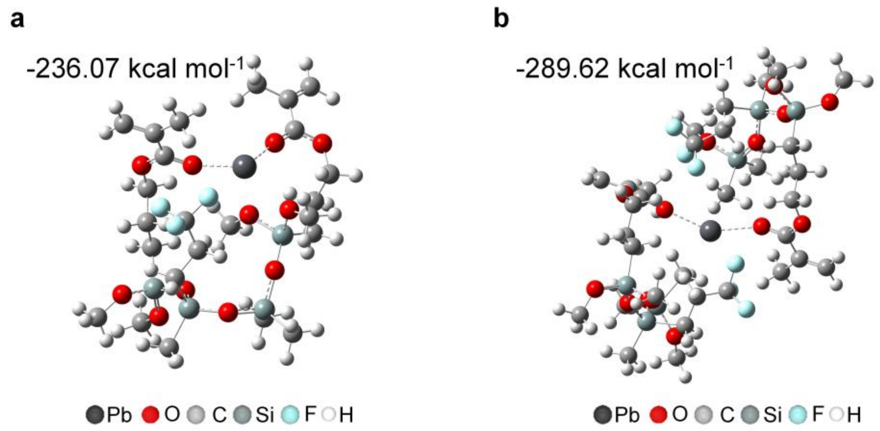

Supplementary Figure 27. Schematic of binding configurations of Pb^{2+} ions with the smallest unit of polymer gel based on the (a) "dimer" model (C=O groups are attached to the same backbone) and (b) "di-monomer" model (two C=O groups not connected by the same main chain). We calculated the binding energies of C=O groups and Pb^{2+} based on density functional theory calculations in **Supplementary Figure 27**. The optimized "dimer" model of C=O groups and Pb^{2+} with the binding energy of -236.07 kcal mol^{-1}, is higher than that of the "di-monomer" model (-289.62 kcal mol^{-1}). The results show that the binding energies in the "di-monomer" model are stronger than in the "dimer" model.

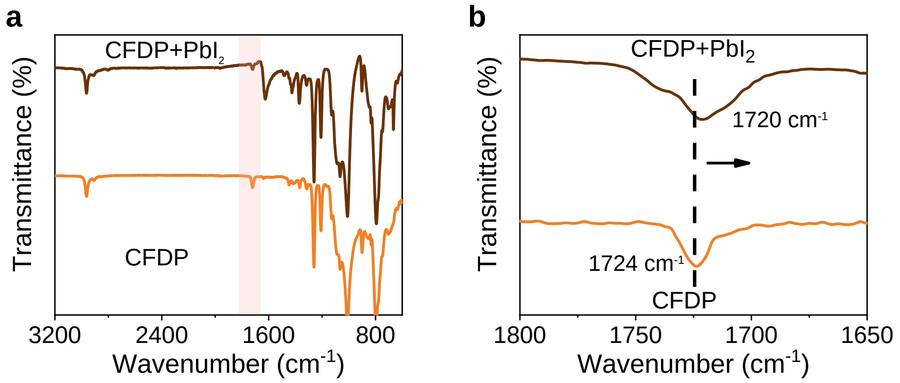

Supplementary Figure 28. (a) FTIR spectra of CFDP and CFDP-PbI_2. (b) Fingerprint region of C=O. The stretching vibration of C=O groups has a redshift when interacting with Pb^{2+} in **Supplementary Figure 28**, suggesting the presence of coordination interaction. In addition, we analyzed the electrostatic potential of the smallest unit in CFDP polymer via the density functional theory (DFT) calculation in **Supplementary Figure 29**. The red area is located at the carbonyl (C=O) position, suggesting a negative electrostatic potential and greater electron density, which is advantageous for coordination with Pb^{2+}.

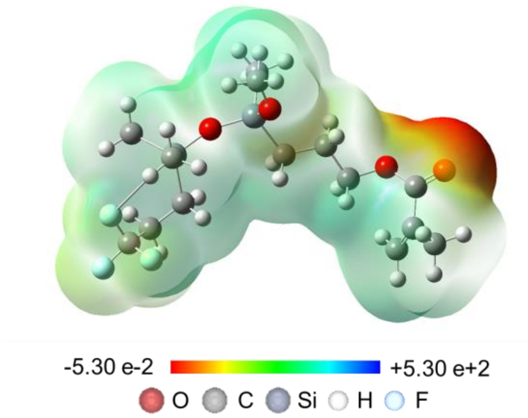

Supplementary Figure 29. The electrostatic potential of the smallest unit in CFDP polymer via DFT calculation.

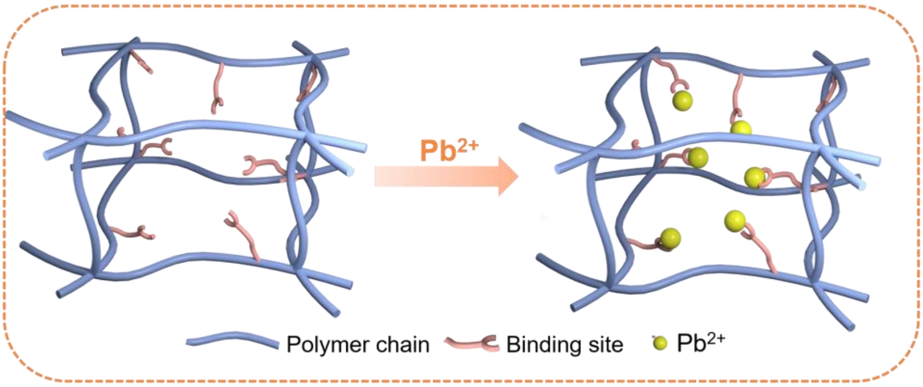

Supplementary Figure 30. Schematic diagram of polymer gel chelated lead ions.

a b

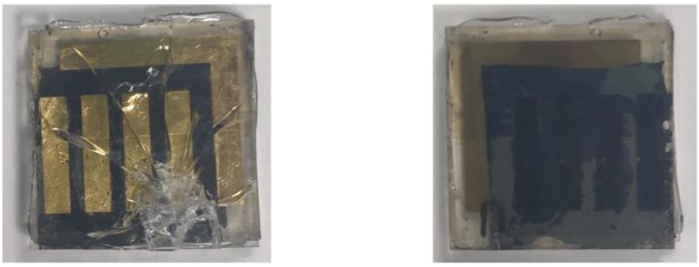

Supplementary Figure 31. Typical process for the preparation of damaged PSCs: (a) the front and (b) the back of a device with POE encapsulation. We further compared the mechanical strength of other encapsulants (POE) via the hail experiment in **Supplementary Figure 31**. We observed that the cover glass on the metal electrode side was seriously damaged, while the glass on the ITO side remained relatively intact.

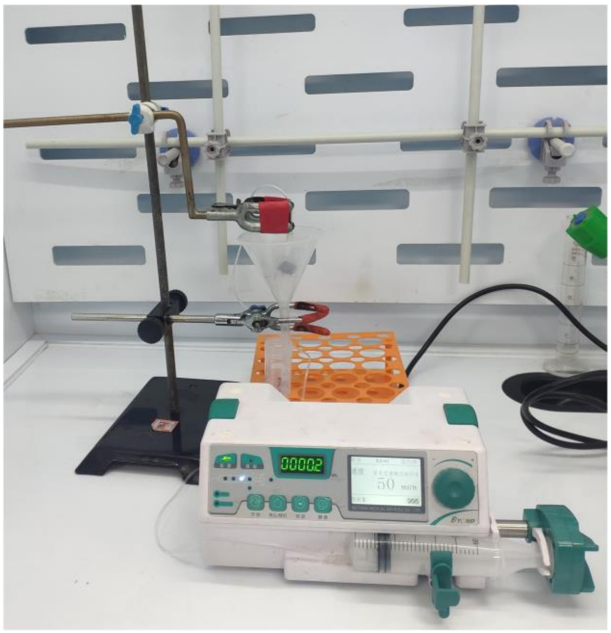

Supplementary Figure 32. Photograph of homemade equipment to study the lead leakage from damaged PSCs under rainfall conditions. The syringe pump facilitated the continuous drop of water on the damaged devices at a rate of 5 mL h^{-1} for 1.5 h. The damaged devices were placed in the funnel at an inclination angle of 30° with respect to the horizon. The contaminated water flowing through the damaged PSCs was collected in a centrifuge tube.

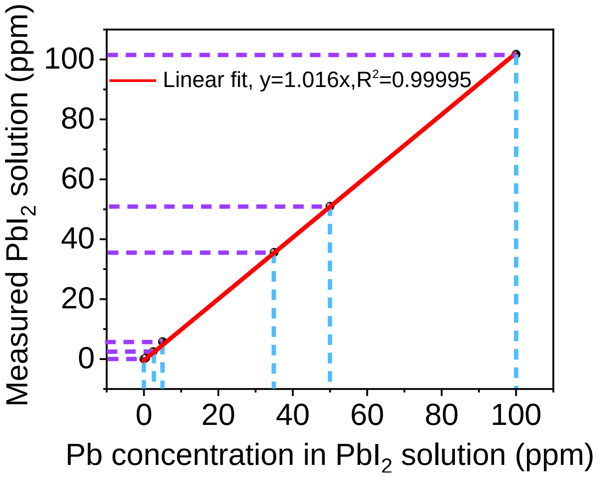

Supplementary Figure 33. The results of accuracy and precision of lead ion detector system.

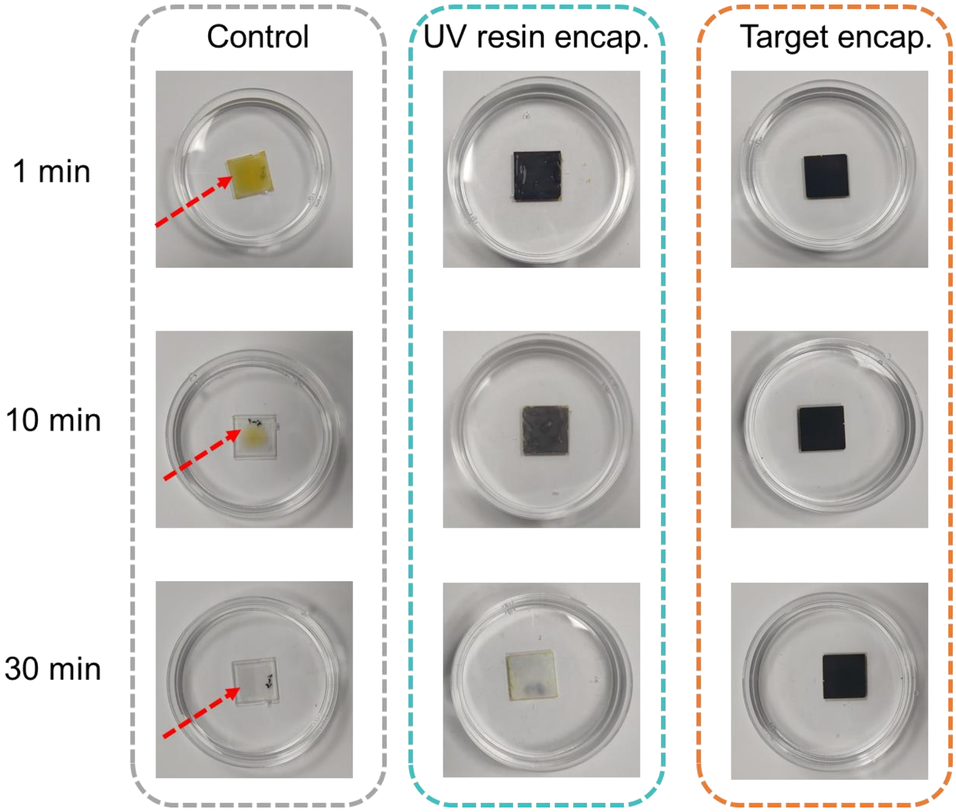

Supplementary Figure 34. Immersing test on the control perovskite film, the perovskite film with UV resin encapsulation, and the perovskite film with target encapsulation, using deionized water with a pH value of 7. We observed that the control perovskite film leaks fast, while the speed of lead leakage with UV resin encapsulation was relatively slow. The perovskite film with target encapsulation remained black phase after 30 min of soaking in water, confirming that CFDP composite gel was excellent in inhibiting lead leakage.

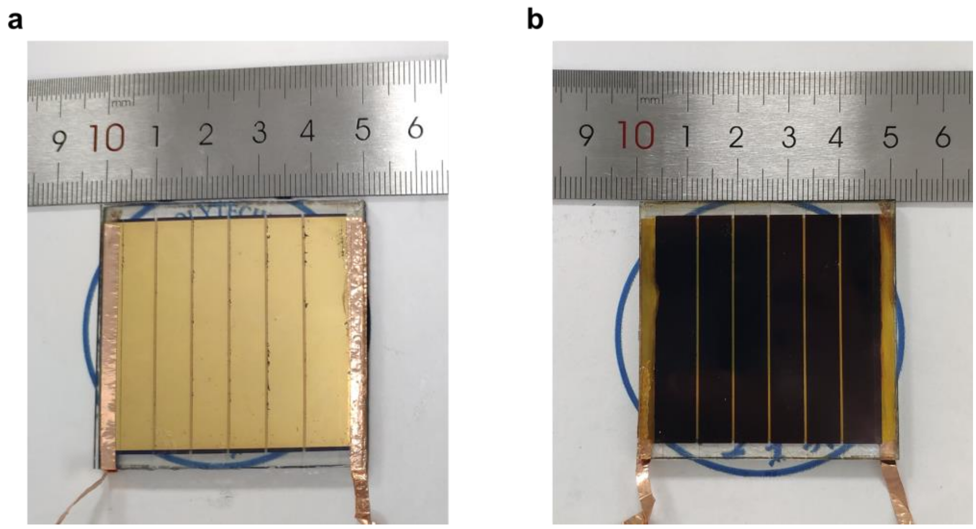

Supplementary Figure 35. Front-side view (a) and back-side view (b) photos of a 5 cm × 5 cm perovskite solar module.

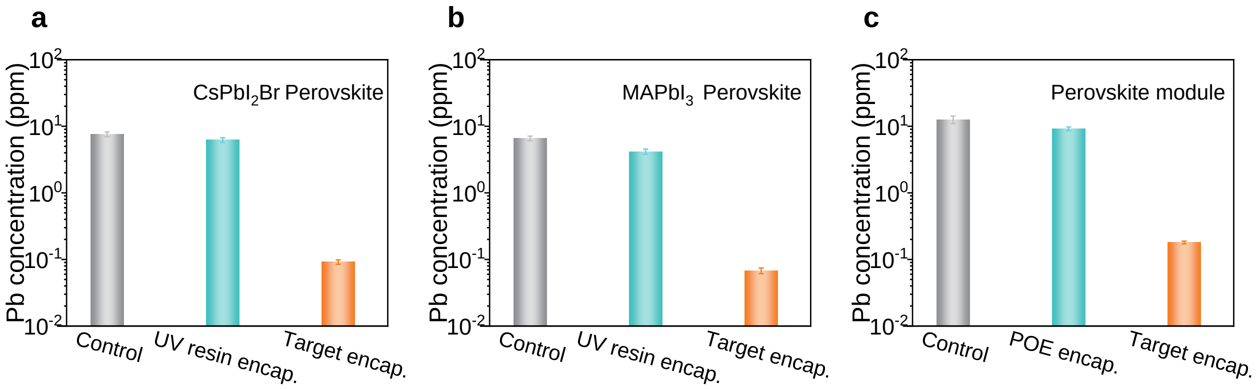

Supplementary Figure 36. Water-dripping test results for the damaged devices of control, UV resin encapsulation, and target encapsulation based on the (a) $CsPbI_2Br$ perovskite, (b) $MAPbI_3$ perovskite, and (c) CsMAFA perovskite module. The error bars represent the standard deviation for three samples.

Supplementary Table 1. The water vapor transmission rate (WVTR) of commonly used EVA and UV resin encapsulants for PSCs compared with CFDP used in this work.[5]

Device	EVA	UV resin	CFDP
WVTR (g/(m^2 day))	28	16	5

Supplementary Table 2. The thermal conductivity of commonly used polymer encapsulants for PSCs in recent reports.

Type	Thermal conductivity ($W\ m^{-1}\ K^{-1}$)
EVA	0.32
POE	0.30
PU	0.25
Paraffin	0.20
PIB	0.20
PO	0.12
UV resin	0.18
CFDP gel (This work)	0.35
CFDP composite gel (This work)	0.51

Supplementary Table 3. Summary of device performance for UV resin encapsulation in a nitrogen glove box at 85 ºC.

Hours	V_{oc} [V]	J_{sc} [mA cm^{-2}]	FF [%]	PCE [%]
0	1.12	23.30	81.34	21.23
240	1.12	22.23	80.94	20.15
480	1.10	22.07	76.23	18.34
720	1.09	21.81	73.59	17.49

Supplementary Table 4. Summary of device performance for target encapsulation in a nitrogen glove box at 85 ºC.

Hours	V_{oc} [V]	J_{sc} [mA cm^{-2}]	FF [%]	PCE [%]
0	1.13	23.61	81.50	21.74
240	1.13	23.29	80.89	21.29
480	1.13	23.21	80.17	21.03
720	1.13	23.10	79.99	20.88

Supplementary Table 5. Summary of device performance for target encapsulation based on $CsPbI_2Br$ perovskite inverted device.

Devices	V_{oc} [V]	J_{sc} [mA cm^{-2}]	FF [%]	PCE [%]
Before	1.18	15.83	77.55	14.48
After	1.18	15.74	76.97	14.30

Supplementary Table 6. Summary of device performance for target encapsulation based on $MAPbI_3$ perovskite normal device.

Devices	V_{oc} [V]	J_{sc} [mA cm^{-2}]	FF [%]	PCE [%]
Before	1.06	21.38	77.04	17.46
After	1.06	21.15	76.57	17.17

Supplementary Table 7. Summary of device performance for target encapsulation based on CsMAFA perovskite modules, effective area is 15.8 cm^2.

Devices	V_{oc} [V]	I_{sc} [mA]	FF [%]	PCE [%]
Before	6.02	62.38	68.11	16.13
After	6.01	61.27	67.90	15.83

Supplementary Table 8. Performance, encapsulation process, and cost summary of perovskite encapsulant.

Type	Adhesion strength (MPa)	Thermal conductivity (W m^{-1} K^{-1})	Stability (damp heat test and thermal cycling test)	Process complexity	Overall processing time (min)	Cost (\$ m^{-2})
UV resin	0.19	0.18	80%/75%	UV light	7	23.87
POE	0.56	0.30	95%/93%	Vacuum hot pressing	15	1.70
CFDP	0.28	0.35	98%/95%	Simple	30	1.91

Supplementary Note 1.

The complexity of the encapsulation process. As shown in **Supplementary Table 8**, the hot pressing under the vacuum environment is required for POE encapsulation. The lamination at 120-150 °C for 15 min is the encapsulation process of POE. In addition, encapsulation with UV resin requires ultraviolet curing equipment. Therefore, the encapsulation process with POE and UV resin is highly dependent on equipment. The target encapsulation is conducted at room temperature. The cross-linking process can be achieved at room temperature and does not require high temperature or UV light to initiate polymerization.

The overall processing time. As shown in **Supplementary Table 8,** the overall processing time of UV resin encapsulation is 7 min, containing the UV exposure time (2 min) and the curing time (5 min). The overall processing time of POE encapsulation is 15 min, which is the time of vacuum hot pressing. The overall processing time of CFDP encapsulation is 30 min, containing the stirring reaction time (5 min) and the curing time (25 min). Because CFDP encapsulation is a room temperature self-crosslinked strategy, the longer waiting time generally doesn't evolve in more processing cost.

The cost analysis of the encapsulation process. As shown in **Supplementary Table 8,** the estimated material cost for the CFDP layer: Combining the estimated usage for ~60 μm thick polymer (~20 g TMPMA m^{-2}; ~450 g F_3 m^{-2}; ~130 g D_4 m^{-2}; ~5 g TMAH m^{-2}; ~10 g DBTDL m^{-2}) and the quotation of raw materials from industrial vendors with large amounts ($5.53 for 1 kg TMPMA; $2.76 for 1 kg F_3; $4.15 for 1 kg D_4; $1.80 for 1 kg TMAH; $1.38 for 1 kg DBTDL. The data was obtained from Alibaba company). The cost could be calculated to be ~1.91 \$ m^{-2}, which is more expensive than commercialized POE (~1.70 \$ m^{-2}) films and cheaper than the price of UV resin (~23.87 \$ m^{-2}).[5] The unit price might be reduced if the CFDP could be industrialized. In addition, encapsulation with POE requires hot pressing equipment and encapsulation with UV resin require UV curing equipment, which will also increase the cost.

Supplementary References

1 Cao, Q. et al. Efficient and stable inverted perovskite solar cells with very high fill factors via incorporation of star-shaped polymer. *Sci. Adv.* **7**, eabg0633, (2021).

2 Huang, Z. et al. Releasing nanocapsules for high-throughput printing of stable perovskite solar cells. *Adv. Energy Mater.* **11**, 2101291, (2021).

3 Han, Y. et al. Degradation observations of encapsulated planar $CH_3NH_3PbI_3$ perovskite solar cells at high temperatures and humidity. *J. Mater. Chem. A* **3**, 8139-8147, (2015).

4 Azmi, R. et al. Damp heat–stable perovskite solar cells with tailored-dimensionality 2D/3D heterojunctions. *Science* **376**, 73-77, (2022).

5 Shi, L. et al. Accelerated lifetime testing of organic–inorganic perovskite solar cells encapsulated by polyisobutylene. *ACS Appl. Mater. Interfaces* **9**, 25073-25081, (2017).
