# Supplementary material for: Room temperature nondestructive encapsulation via self-crosslinked fluorosilicone polymer enables damp heat-stable sustainable perovskite solar cells
Source: Nat Commun. 2023 Mar 11;14:1342. doi: 10.1038/s41467-023-36918-x (PMC10008636; doi:10.1038/s41467-023-36918-x)
Supplement: Supplementary file 3 — Solar Cells Reporting Summary [file 41467_2023_36918_MOESM3_ESM.pdf]

nature research

Corresponding author(s): Xuanhua Li

# Solar Cells Reporting Summary

Nature Research wishes to improve the reproducibility of the work that we publish. This form is intended for publication with all accepted papers reporting the characterization of photovoltaic devices and provides structure for consistency and transparency in reporting. Some list items might not apply to an individual manuscript, but all fields must be completed for clarity.

For further information on Nature Research policies, including our data availability policy, see Authors & Referees.

## Experimental design

**Please check: are the following details reported in the manuscript?**

1. Dimensions

| Item | Yes/No | Details |
|---|---|---|
| Area of the tested solar cells | ☒ Yes ☐ No | Methods section, device fabrication. |
| Method used to determine the device area | ☒ Yes ☐ No | Methods section, device fabrication. |

2. Current-voltage characterization

| Item | Yes/No | Details |
|---|---|---|
| Current density-voltage (J-V) plots in both forward and backward direction | ☐ Yes ☒ No | The plots in backward direction are provided in the manuscript. Figure 3d. |
| Voltage scan conditions<br>*For instance: scan direction, speed, dwell times* | ☒ Yes ☐ No | The scanning speed is 0.1 V/s and the bias ranges is from -0.1 V to 1.2 V under reverse voltage scan. Methods section, device characterization. |
| Test environment<br>*For instance: characterization temperature, in air or in glove box* | ☒ Yes ☐ No | All J-V results are measured in a nitrogen filled glove box. Methods section, device characterization. |
| Protocol for preconditioning of the device before its characterization | ☐ Yes ☒ No | No preconditioning is used in this work. |
| Stability of the J-V characteristic<br>*Verified with time evolution of the maximum power point or with the photocurrent at maximum power point; see ref. 7 for details.* | ☐ Yes ☒ No | No relevant to our study. |

3. Hysteresis or any other unusual behaviour

| Item | Yes/No | Details |
|---|---|---|
| Description of the unusual behaviour observed during the characterization | ☐ Yes ☒ No | No relevant to our study. |
| Related experimental data | ☐ Yes ☒ No | No relevant to our study. |

4. Efficiency

| Item | Yes/No | Details |
|---|---|---|
| External quantum efficiency (EQE) or incident photons to current efficiency (IPCE) | ☐ Yes ☒ No | No relevant to our study. |
| A comparison between the integrated response under the standard reference spectrum and the response measure under the simulator | ☐ Yes ☒ No | No relevant to our study. |
| For tandem solar cells, the bias illumination and bias voltage used for each subcell | ☐ Yes ☒ No | No tandem solar cells are present in this paper. |

5. Calibration

| Item | Yes/No | Details |
|---|---|---|
| Light source and reference cell or sensor used for the characterization | ☒ Yes ☐ No | J-V characteristics of PSCs were performed via a keithley 2420 sourcemeter under AM 1.5 G illumination (Newport 94023A Oriel Sol3A, Class AAA, 100 mW cm−2 calibrated with a silicon reference cell (Hamamatsu S1133)). Methods section, device characterization. |


November 2017

Confirmation that the reference cell was calibrated and certified

☒ Yes ☐ No

The reference cells were calibrated and certified.

Calculation of spectral mismatch between the reference cell and the devices under test

☐ Yes ☒ No

The light spectrum used for the measurements matches well with the reference silicon cell, we do not calculated the spectral mismatch between the reference cell and the tested cells

6. Mask/aperture

Size of the mask/aperture used during testing

☐ Yes ☒ No

No shadow mask is used.

Variation of the measured short-circuit current density with the mask/aperture area

☐ Yes ☒ No

We have not measured short-circuit current density with aperture area.

7. Performance certification

Identity of the independent certification laboratory that confirmed the photovoltaic performance

☐ Yes ☒ No

Solar cell efficiency is not certified.

A copy of any certificate(s)
*Provide in Supplementary Information*

☐ Yes ☒ No

No certificate(s).

8. Statistics

Number of solar cells tested

☒ Yes ☐ No

Supplementary Figure 21.

Statistical analysis of the device performance

☒ Yes ☐ No

Supplementary Figure 21.

9. Long-term stability analysis

Type of analysis, bias conditions and environmental conditions
*For instance: illumination type, temperature, atmosphere humidity, encapsulation method, preconditioning temperature*

☒ Yes ☐ No

Figure 3e-f and Figure 5, Supplementary Figure 25-26. Methods section, Stability test.
